# Pentacyclic triterpenoid-rich fraction of the Hardy kiwi (*Actinidia arguta*) improves brain dysfunction in high fat diet-induced obese mice

**DOI:** 10.1038/s41598-020-62810-5

**Published:** 2020-04-01

**Authors:** Jeong Su Ha, Jin Yong Kang, Jeong Eun Kang, Seon Kyeong Park, Jong Min Kim, Chul-Woo Kim, Sung-Il Oh, Uk Lee, Dae-Ok Kim, Ho Jin Heo

**Affiliations:** 1 Division of Applied Life Science (BK21 plus), Institute of Agriculture and Life Science, Gyeongsang National University, Jinju, 52828 Republic of Korea; 2 Division of Special Forest Products, National Institute of Forest Science, Suwon, 16631 Republic of Korea; 3 Department of Food Science and Biotechnology, Kyung Hee University, Yongin, 17104 Republic of Korea

**Keywords:** Cognitive control, Alzheimer's disease

## Abstract

This study was performed to investigate the effect of the chloroform fraction from *Actinidia arguta* (CFAA) on cognitive dysfunction in a C57BL/6 mouse model fed a high-fat diet (HFD) for 12 weeks. The CFAA has the protective effect on high glucose-induced neurotoxicity in MC-IXC cell (neuroblastoma cell line). In a C57BL/6 mouse model fed a HFD for 12 weeks, the improved glucose tolerance and cognitive dysfunction were observed in a group ingesting CFAA. In the brain tissue analysis, the impaired cholinergic, antioxidant system and mitochondria functions were improved in the CFAA group. In addition, in a molecular biology study, it was observed that CFAA improves HFD-induced abnormal insulin signaling such as increase of IRS phosphorylation at serine residues and reduction of Akt phosphorylation caused by the increase of JNK phosphorylation and then inhibited apoptosis. In the UPLC Q-TOF/MS analysis, pentacyclic triterpenoids such as asiatic acid (AA), madecassic acid (MA) were identified in CFAA as main compounds. Therefore, these results propose that *Actinidia arguta* rich in pentacyclic triterpenoids may be effective as preventive matter a therapeutic strategy to improve neurodegenerative disease caused by HFD.

## Introduction

A western diet and sedentary life have caused an increase in surplus energy in the body, and surplus energy causes obesity. Excessively accumulated fat, one of the main features of obesity, promotes oxidative stress by increasing the production of free radicals such as reactive oxygen species (ROS) and reactive nitrogen species (RNS), resulting in an imbalance in the body’s antioxidant system^1^. The sustained oxidative stress induces the inhibition of glucose utilization in muscle and adipose tissue, so the complications of obesity include hyperlipidemia, cardiovascular disease, fatty liver, degenerative arthritis, heart disease and type 2 diabetes mellitus (T2DM)^2^.

In numerous past epidemiological studies, it became clear that T2DM is a risk factor for developing Alzheimer’s disease (AD), in addition, T2DM-related conditions, including obesity, hyperinsulinemia, and metabolic syndrome, may also be risk factors for AD^3^. AD is the most common form of dementia, accounting for 60–70% of all cases, and the pathological features of AD include amyloid plaques constituted by amyloid beta as the main substance and neurofibrillary tangles aggregated of hyperphosphorylated tau protein, thus, it was proposed that amyloid plaques and neurofibrillary tangles cause AD^4^. In a previous study, it was confirmed that insulin resistance in the brain caused by T2DM elevates the production of Aβ oligomers and hyperphosphorylation of tau by increasing GSK-3β and β-secretase^5^.

Diets high in fruits and vegetables are widely recommended for their health-promoting properties, and various epidemiological and clinical studies have proved the health benefits of fruits and vegetables, and has also been confirmed that fruits and vegetables can improve and prevent various diseases^6^.

*Actinidia arguta* (*A. arguta*), the hardy kiwi, is widespread in northeastern Asia and the fruits of *A. arguta*, called a “healthy fruit,” contain a variety of vitamins, polyphenols, some minerals, amino acids and fatty acids, in addition, it is also reported that the fruit of *A. arguta* has excellent antioxidant activity as well as anti-inflammatory activity^7^. In addition, according to the research of Kurakane *et al*.^8^, the polyphenols of *A. arguta* fruit improved the glucose tolerance of diabetic mice, and we have confirmed excellent α-glucosidase and α-amylase inhibitory activity in the chloroform fraction from *A. arguta* (CFAA) (Supplementary Fig. S1). Compared with acarbose as a positive control, CFAA had superior inhibitory activity on α-glucosidase and α-amylase, which are both enzymes involved in the digestion of carbohydrates, and the α-glucosidase inhibitory activity of CFAA was more effective than acarbose. Already, various natural α-glucosidase and α-amylase inhibitors from plant sources have been reported, and it was reported that the inhibition of α-glucosidase and α-amylase can significantly reduce the postprandial increase in blood glucose and therefore can be an important strategy in the management of blood glucose level in type 2 diabetes and borderline patients^9^. With these various results, we hypothesised that CFAA may be effective in improving cognitive decline in HFD-induced insulin-resistant mice, and CFAA has interesting physiological active compounds of *A. arguta*. Therefore, this study was performed to investigate the improvement effect of CFAA in HFD-induced insulin-resistant mice and identify the main compounds of CFAA.

## Materials and methods

### Materials

Thiobarbituric acid (TBA), metaphosphoric acid, 2,7-dichlorodihydrofluorescein diacetate (DCF-DA), 3-(4,5-dimethylthiazol-2-yl)-2,5-diphenyl tetra-zolium bromide (MTT), 5,5,6,6-tetrachloro-1,1,3,3-tetraethylbenzimidazolylcarbocyanine iodide (JC-1), lactate dehydrogenase (LDH) assay kit, superoxide dismutase (SOD) determination kit, adenosine triphosphate (ATP) bioluminescence assay kit and all other chemicals used were purchased from Sigma-Aldrich Chemical Co. (St. Louis, MO, USA). A total glutathione (GSH) kit was purchased from Enzo Life Science Inc. (Farmingdale, NY, USA).

### Preparation of samples

The *A. arguta* fruit (cultivar: Autumn Sense) was obtained from the National Institute of Forest Science (Suwon, Korea) in September 2013. *A. arguta* was extracted in 40% ethanol at 40 °C for 2 h. The extract obtained was filtered, concentrated using a rotary evaporator, and re-dissolved with distilled water. Then it was consecutively partitioned using a separatory funnel with solvents including *n*-hexane, chloroform, and ethyl acetate. Lastly, the CFAA obtained was evaporated and lyophilized, and stored at −20 °C until used.

### Cell culture and neuroprotection

Human neuroblastoma MC-IXC cells (ATCC^®^-CRL-2270^TM^) were cultured in minimal essential medium containing 10% FBS, 50 units/mL penicillin and 100 μg/mL streptomycin in conditions of 5% CO_2_ and 37 °C.

Intracellular oxidative stress was measured by a DCF-DA assay. After seeding cells at 1 × 10^4^/well and incubating for 24 hours, CFAA and vitamin C were pretreated in cells for 3 h. Next, 50 mM high glucose (HG) was continually treated for 24 h, and then was treated with 10 μM DCF-DA dissolved in PBS for 1 h. The level of intracellular dichlorofluorescein production was measured with a fluorescence microplate reader (Infinite 200, Tecan Co., San Jose, CA, USA).

Cell viability was measured by a MTT reduction assay. After the sample and high glucose (HG) were treated in the same concentration and time, MTT solution (5 mg/mL) was treated for 3 h. Subsequently, MTT formazan crystals were dissolved by adding DMSO. The absorbance was measured by a microplate reader with a 570 nm test wavelength and 690 nm reference wavelength.

To examine the protective effect on the neuronal cell membrane, the quantity of lactate dehydrogenase (LDH) released was determined. After the sample and HG were treated in the same concentration and time, the amounts of LDH released into the medium were determined using a commercial kit.

### Animals and *in vivo* design

All animal experiments followed the guidelines established by the Animal Care and Use Committee of Gyeongsang National University (certificate: GNU-160531-M0026) approved by the Animal and Plant Quarantine Agency in Ministry of Agriculture, Food and Rural Affairs, Republic of Korea. C57BL/6 mice (4 weeks old, male) were purchased from Samtako (Osan, Korea), and were housed with free access to water and feed in a room under maintained conditions (12 h light/dark cycle, 22 ± 2 °C, and 55% humidity). The mice were randomly assigned to four groups (n = 12): normal chow diet (NC, including protein (20 kcal%/g), carbohydrate (70 kcal%/g) and fat (10 kcal%/g), 3.85 kcal/g), high-fat diet (HFD, including protein (20 kcal%/g), carbohydrate (20 kcal%/g) and fat (60 kcal%/g), 5.24 kcal/g), CFAA 20 and 40. After 12 weeks of NC and HFD, the CFAA groups were intragastrically fed purified water containing CFAA (20 and 40 mg/kg of body weight, respectively), whereas the NC and HFD groups were fed the same volume of purified water for 4 weeks.

### Behavioral tests

Spontaneous alternation was performed in a Y-maze consisting of white-painted plastic material. This test is performed once without training, and mice were placed on the end of one arm, and the moving routes of mice were measured by using the SMART video-tracking system (Smart v3.0 Panlab SL, Barcelona, Spain) for 8 min. Successful alternation was defined as consecutive entries into the three arms.

A passive avoidance test was performed for 2 days on an apparatus consisting of an illuminated room, a dark room, and an electrified wire mesh floor. On first day, the mice were placed in the bright room and allowed to enter the dark room. An inescapable electronic shock (0.5 mA) was provided to the mice for 3 s when their hind paws entered the dark room. Next day, the mice were placed in the bright room again, and the latency time of entrance into the dark room was recorded (maximum limit latency time: 300 s).

A Morris water maze (MWM) test was performed, somewhat modified from Morris’s study^10^. The apparatus consisted of a stainless steel circular pool randomly divided into quadrants. An escape platform was placed in the W zone. On training days, the mice were allowed to swim and the latency time was recorded (maximum limit latency time: 60 s) using the SMART video-tracking system. The training trial was subjected to swimming to escape (four times with an interval of 30 min repeatedly per day) during 4 days. A probe trial was examined without the platform, and the retention time in the W zone was recorded.

### Intraperitoneal glucose tolerance test (IPGTT)

After *in vivo* tests, the IPGTT was performed. All mice were fasted for 8 h, and injected intraperitoneally (i.p.) with D-glucose (2 g/kg of body weight) in sterile saline (0.9% NaCl). Blood glucose obtained from the caudal vessels was measured at 0, 15, 30, 60, 90 and 120 min using an Accu-Chek glucose meter (Roche Diagnostics Australia Pty. Ltd., Castle Hill, Australia).

### Sampling of serum and organs

To investigate biochemical changes, the mice were sacrificed by CO_2_ inhalation to get blood and tissues (brain and liver). The blood was collected from the abdominal aorta into a heparin tube. The blood was centrifuged at 10,000 g for 10 min, and serum obtained from blood was immediately analyzed. The tissues were homogenized in a bullet blender (Nextadvance Inc., Averill Park, NY, USA) with ice-cold 10-fold volumes of PBS and 20-fold volumes of 5% metaphosphoric acid. The protein concentration of brain and liver was measured using the Bradford protein assay.

### Serum analysis

The glutamic oxaloacetic transaminase (GOT), glutamine pyruvic transaminase (GPT), LDH, total cholesterol (TCHO), triglyceride (TG) and high density lipoprotein cholesterol (HDLC) levels in the serum were measured using clinical chemistry analyzer (Fuji dri-chem 4000i; Fuji film Co., Tokyo, Japan).

### Cholinergic system in brain tissue

Acetylcholine (ACh) contents and acetylcholinesterase (AChE) activity in the brain tissue were measured as a colorimetric assay (*n* = 7)^11^. The tissue was homogenized with 10-fold volumes of PBS and centrifuged at 14,000 g for 30 min. Supernatant was added to a 20 μL alkaline hydroxylamine reagent (2 M hydroxylamine-hydrochloride and 3.5 N sodium hydroxide, 1:1 ratio), and the mixture was incubated at room temperature for 1 h. Then the reactant was treated with 0.5 N hydrochloride and 0.3 M iron chloride hexahydrate, and absorbance was immediately measured at 540 nm to get ACh contents.

To measure AChE activity (*n* = 7), supernatant was added to 50 mM sodium phosphate buffer (pH 7.4), and the mixture was incubated at 37 °C for 15 min. Then the reactant was treated with 70 μL Ellman’s reaction mixture [0.5 mM acetylthiocholine and 1 mM 5,5’dithiobis (2-nitrobenzoic acid) in a 50 mM sodium phosphate buffer (pH 7.4)] and incubated at 37 °C for 10 min. The absorbance at 405 nm was measured^11^.

### Antioxidant system in brain and liver tissues

To measure SOD contents, the tissues were homogenized with PBS and centrifuged at 400 g for 10 min to get the pellets. The obtained pellets were mixed with cell extraction buffer [10% SOD buffer, 0.4% (v/v) Triton X-100, and 200 μM phenylmethanesulfonyl fluoride in distilled water], and centrifuged at 10,000 g for 10 min. The supernatants were determined using the protocol provided in the SOD assay kit.

To measure oxidized glutathione (GSH)/total GSH ratio, the tissues were homogenized with 5% metaphosphoric acid and centrifuged at 14,000 g for 15 min to get the supernatants. Some of the supernatants were treated with 2 M 4-vinylpyridine at room temperature for 1 h to get the oxidized GSH. Both supernatants were determined using the protocol provided in the GSH detection kit.

To measure malondialdehyde (MDA) contents, the tissues were homogenized with PBS and centrifuged at 6,000 g for 30 min to get the supernatants. The supernatants were treated with 1% phosphoric acid (v/v) and 0.67% TBA (w/v) solution before they were incubated at 95 °C for 1 h. The reactant was centrifuged at 5,000 g for 1 min, and the absorbance was immediately measured at 532 nm. The protein concentration was determined using the Bradford protein assay.

### Preparation of mitochondria and mitochondrial activity

The preparation of isolated mitochondria was conducted as described in the method^12^. Brain tissue was homogenized with 10 volumes of isolation buffer (20 mM HEPES sodium salt, 75 mM sucrose, 0.1% bovine serum albumin, 215 mM mannitol, pH 7.2) including 1 mM ethylene-glycol-bis(2-aminoethylether)-*N,N,N’,N’*-tetraacetic acid (EGTA), and centrifuged at 1,300 g for 10 min to remove unbroken cells and nuclei. The supernatant was placed in new tubes and re-centrifuged at 13,000 g for 10 min to get the pellets. The obtained pellets were treated with isolation buffer including 0.1% digitonin in DMSO to disrupt the synaptosomes. After 5 min, isolation buffer including 1 mM EGTA was added to the mixtures before they were centrifuged at 13,000 g for 15 min. The obtained pellets were resuspended with isolation buffer and were centrifuged at 10,000 g for 10 min to get the mitochondrial pellets. Finally, the pellets were resuspended with isolation buffer to get the isolated mitochondrial solution (a final protein concentration of approximately 10 mg/mL) and used.

The ROS production in mitochondria was determined using a respiration buffer (125 mM potassium chloride, 20 mM HEPES, 2.5 mM malate, 2 mM potassium phosphate, 5 mM pyruvate, 1 mM magnesium chloride and 500 μM EGTA, pH 7.0). In brief, a mitochondrial solution (0.8 mg/mL of protein concentration) was incubated with 25 μM DCF-DA in respiration buffer at room temperature for 20 min, and reactant was measured.

The mitochondrial membrane potential (MMP) in mitochondria was determined using an assay buffer (mitochondrial isolation buffer including 5 mM pyruvate and 5 mM malate). In brief, mitochondrial solution (1.2 mg/ml of protein concentration) was incubated with 1 μM JC-1 in assay buffer at room temperature for 20 min, and reactant was measured.

The adenosine triphosphate (ATP) in mitochondria was determined using an ENLITEN ATP assay system bioluminescence detection kit (Promega Corporation Inc., Madison, WI, USA) according to the provided protocol. The ATP level was calculated according to a standard curve.

### Western blot analysis

10 μL equal amounts of soluble protein through relative quantification obtained from brain tissue were separated by SDS-PAGE and transferred onto a polyvinylidene difluoride (PVDF) membrane (Millipore, Billerica, MA, USA). Immunoblotting was carried out with primary antibodies (1:1,000) for p-JNK (Thr 183/Tyr 185); sc‐6254, p-IRS (Ser 307); sc‐33956, p-Akt (Ser 473); sc‐514032, p-tau (Ser 404); sc‐12952, Bax; sc-2772, Cytochrome c; sc‐13560, AChE; sc-373901 and β-actin; sc‐69879. After leaving overnight, a secondary antibody (1:2,000) [anti-rabbit (7074 S) and anti-mouse (7076 S)] was reacted for 1 h, and visualized using an chemiluminescence (ECL) reagent (Bionote, Hwaseong, Korea) by Chemi-doc (Invitrogen Co., Carlsbad, CA, USA). Data analysis was done with Image. J., and calculated with density of target protein/density of β-actin as a loading control.

### UPLC Q-TOF/MS analysis

The compound analysis of CFAA was carried out with a UPLC-Q-TOF MS (Waters, Milford, MA, USA) equipped with Acquity UPLC BEH C_18_ columns (100 × 2.1 mm, 1.7 μm, Waters, Milford, MA, USA). Mobile phase A was water containing 0.1% formic acid and mobile phase B was acetonitrile containing 0.1% formic acid, and the solvent gradient conditions were as follows: a gradient elution of 99% A/1% B to 1%A/99%B at 0–20 min in a flow rate of 0.4 ml/min. To obtain MS^2^ data, ionization was operated with a negative electrospray (ESI) mode, and the optimized conditions were follows: ramp collision energy, 40–60 eV; capillary voltage, 3 kV; desolvation temperature, 350 °C; pressure of nebulizer, 40 psi; fragmentor, 175 V; cone voltage, 40 V; mass range, 50–1,200 m/z; oven temperature, 40 °C. All data were acquired and processed using UNIFI® v1.8.1 software (Waters, Milford, MA, USA).

### Statistical analysis

The results were expressed as mean ± SD, and analyzed by one-way analysis of variance (ANOVA) followed by Duncan’s multiple range test (*p* < 0.05) with the help of the SAS program (ver. 9.1, SAS Institute, Cary, NC, USA).

## Results

### Neuroprotective effect

**Figure 1:**
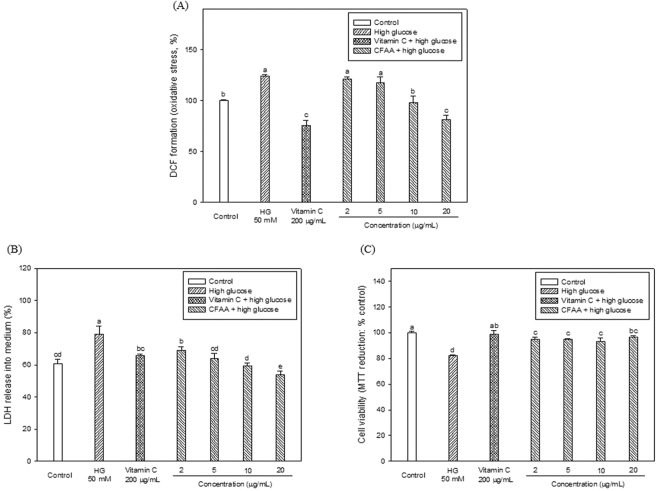
Effect of chloroform fraction from *Actinidia arguta* (CFAA) on ROS production (**A**), LDH release into medium (**B**) and cell viability (**C**) in HG-induced neurotoxicity. Data were analyzed using ANOVA with Duncan’s SAS and expressed as mean ± SD (*n* = 3). Data were statistically considered at *p* < 0.05, and different small letters represent statistical difference.

The ROS production caused by HG was measured using a DCF-DA assay, and the effect of CFAA is shown in Fig. 1A. The HG-treated group showed a significant increase in ROS production (123.84%) compared to the NC group (100.00%). On the other hand, the CFAA-treated groups (10 and 20 μg/mL concentrations) showed a significant decline in ROS production to 97.99% (*p* < 0.05) and 81.50% (*p* < 0.05), respectively compared to HG group. In particular, the CFAA-treated at 20 μg/mL concentration, unlike other concentrations of CFAA (*p* < 0.05), showed an effect of ROS reduction similar to the vitamin C group (75.81%, *p* > 0.05). To evaluate the protective effect of CFAA on the neuronal membrane, the quantity of LDH released into medium was measured, as shown in Fig. 1B. The HG-treated group showed a significant increase in LDH quantity (79.02%) in comparison with the NC group (60.87%, *p* < 0.05). In contrast, the CFAA-treated groups showed a significantly decreased LDH quantity compared with that of the HG-treated group at all concentrations (*p* < 0.05). In particular, the CFAA-treated groups (10 and 20 μg/mL concentrations) showed a significantly lower LDH quantity compared with that of the vitamin C group (65.70%, *p* < 0.05).

Cell viability with HG-induced neurotoxicity was measured using an MTT reduction assay, and effect of CFAA was evaluated (Fig. 1C). The HG-treated group showed a low cell viability (82.12%) compared with that of the NC group (100.00%, *p* < 0.05), whereas, the CFAA-treated groups showed increased cell viability but not as much as the control group (*p* < 0.05) compared with that of the HG-treated group at all concentrations (*p* < 0.05), but not as much as the control group (*p* < 0.05).

### Fasting blood glucose and IPGTT

**Figure 2:**
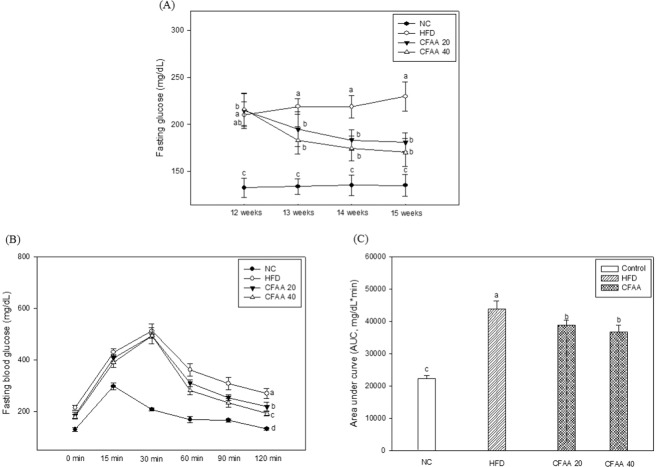
Effect of chloroform fraction from *Actinidia arguta* (CFAA) on fasting glucose level (**A**), intraperitoneal glucose tolerance test (IPGTT) (**B**) in HFD-induced diabetic mice and Area under the curve (AUC) (**C**) in IPGTT. Data were analyzed using ANOVA with Duncan’s SAS and expressed as mean ± SD (*n* = 7). Data were statistically considered at *p* < 0.05, and different small letters represent statistical difference. In (**A**,**B**) statistical difference in the result of same time zone was analyzed.

The change of fasting blood glucose during the intake period (4 weeks) of CFAA is shown in Fig. 2A. Before the intake of CFAA, both the HFD group (*p* < 0.05) and CFAA groups (20 and 40 mg/kg of body weight, *p* < 0.05) showed significantly higher fasting blood glucose compared with the NC group (132.86 mg/dL). During the intake period, the fasting blood glucose of the CFAA groups (20 and 40 mg/kg of body weight) significantly decreased compared with the HFD group. After ending the CFAA intake period, the fasting blood glucose of the HFD group increased to 229.71 mg/dL, whereas the CFAA 20 and 40 groups decreased to 181.14 mg/dL (*p* < 0.05) and 170.43 mg/dL (*p* < 0.05), respectively. In addition, in the results of IPGTT, it was observed that glucose tolerance improved in the CFAA groups compared with the HFD group (Fig. 2B). In the calculated AUC for identify overall differences in glucose excursion between groups, it was confirmed that the AUC of the HFD group was higher than other groups (*p* < 0.05), and the ACU of CFAA 20 and 40 group were decreased than the HFD group but not as much as the NC group (Fig. 2C) (*p* < 0.05).

### Behavioral tests

**Figure 3:**
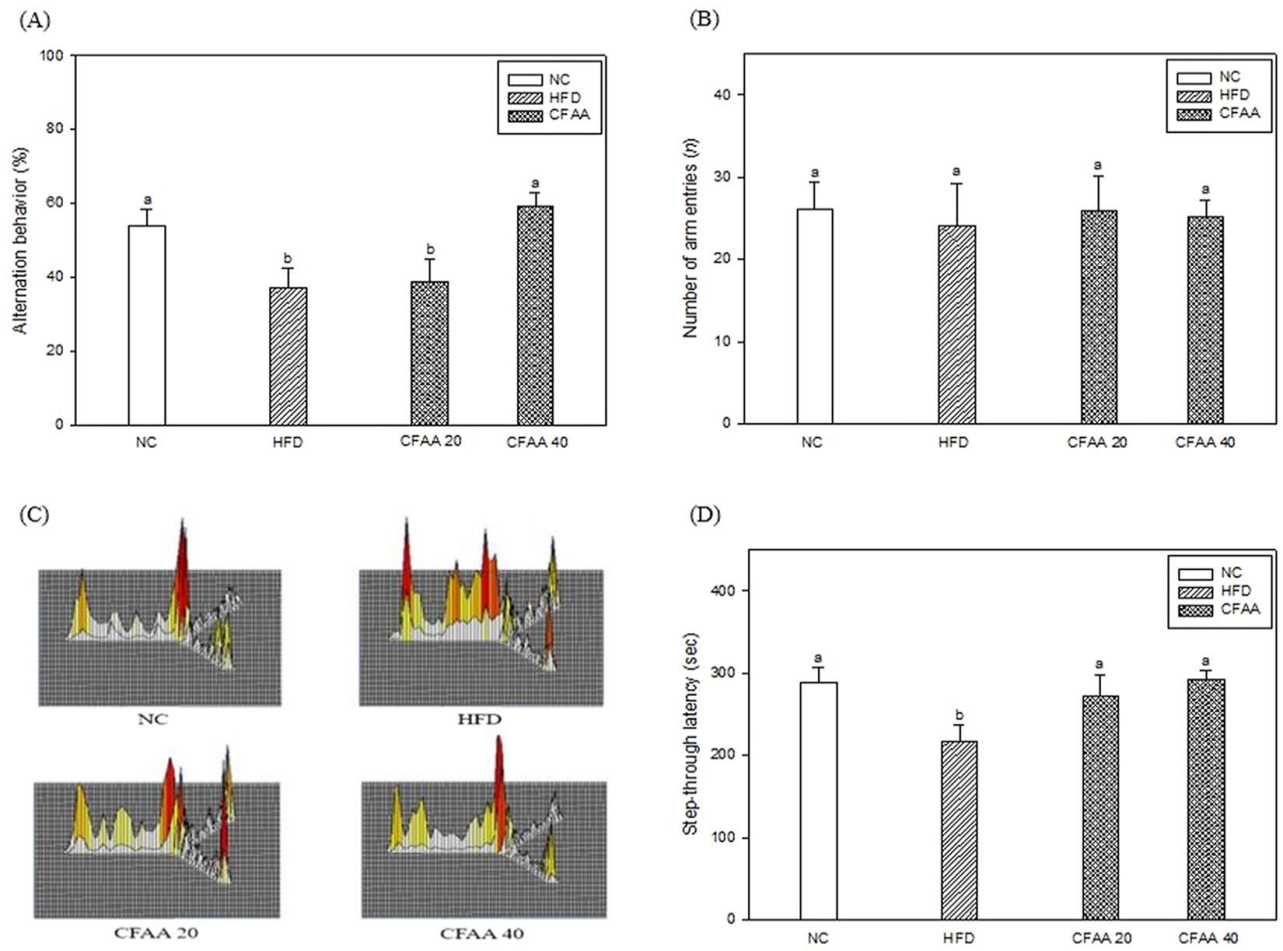
Effect of chloroform fraction from *Actinidia arguta* (CFAA) in high-fat diet-induced diabetic mice on alternation behavior (**A**), number of arm enteries (**B**), path tracing of each groups in the Y-maze (**C**) and step-through latency (**D**). Data were analyzed using ANOVA with Duncan’s SAS and expressed as mean ± SD (*n* = 7). Data were statistically considered at *p* < 0.05, and different small letters represent statistical difference.

Behavioral tests including Y-maze, passive avoidance and MWM tests were performed to assess the effect of CFAA on the HFD-induced cognitive defect model. In the results of spatial cognitive ability by using the Y-maze, as shown in Fig. 3A, the alternation behavior of CFAA 20 group (38.79%, *p* > 0.05) did not increase significantly compared to HFD group. However, the CFAA 40 group appeared to have increased alternation behavior with 59.31% (*p* > 0.05), significantly similar to the NC group. In contrast, there was no statistical difference in the number of arm entries in all groups (Fig. 3B) (*p* > 0.05). These data are consistent where the moving routes of the HFD group were imbalanced, whereas the CFAA 40 group showed a similar form to that of the NC group (Fig. 3C).

Short-term memory ability was measured by using a passive avoidance test, as shown in Fig. 3D. The HFD group showed a significantly low step-through latency time of 217.40 s compared with the NC group (289.00 s, *p* < 0.05), whereas the CFAA groups (20 and 40 mg/kg of body weight) showed improved step-through latency time of 271.80 (*p* < 0.05) and 292.80 s (*p* < 0.05) compared with HFD group, respectively.

**Figure 4:**
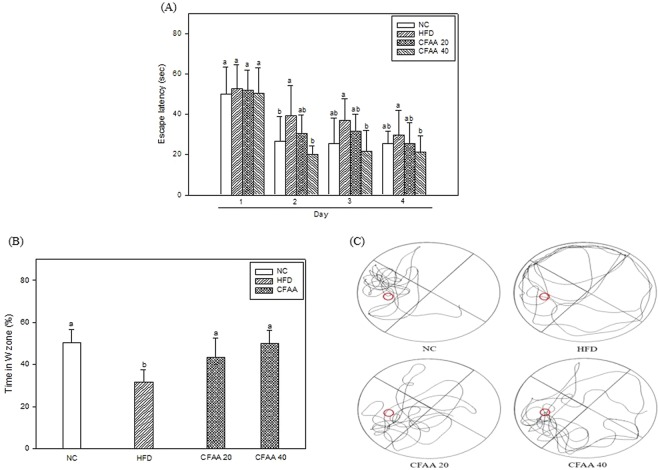
Effect of chloroform fraction from *Actinidia arguta* (CFAA) in high-fat diet-induced diabetic mice on escape latency (**A**), N zone crossings of probe trial sessions (**B**) and path tracing of each groups in the probe trial (**C**). Data were analyzed using ANOVA with Duncan’s SAS and expressed as mean ± SD (*n* = 7). Data were statistically considered at *p* < 0.05, and different small letters represent statistical difference.

Long-term memory and spatial navigation abilities were measured by using the MWM test, as shown in Fig. 4. In the training session, as the training was repeated, the escape time decreased in all groups (Fig. 4A), the decreased escape latency time at 1 to 4 day is most higher in the HFD group (HFD group 52.88 -> 29.84 s) than other groups (NC group; 50.08 -> 25.57 s, CFAA 20; 51.92 -> 25.47 s, CFAA 40; 50.29 -> 21.30 s). In the probe test (Fig. 4B), the retention time in the W zone for the HFD group decreased to 31.60% compared with the NC group (50.44%, *p* < 0.05), whereas the time for the CFAA groups (20 and 40 mg/kg of body weight) significantly increased to 43.54 (*p* < 0.05) and 49.90% (*p* < 0.05) compared with HFD group, respectively. As shown in Fig. 4C, the CFAA groups had more path tracing in the W zone compared to the HFD group.

### Serum analysis

Table 1 showed the effect of CFAA on serum biochemical changes in the HFD-induced mice. All serum biochemical were increased in the HFD group compared with NC group (*p* < 0.05). On the other hand, the CFAA groups were tendency to decrease in all biochemical compared with the HFD group. In particular, in the CFAA 40 group, the decrease of biochemicals such as GOT (p < 0.05), GPT (p < 0.05), LDH (p < 0.05) and TCHO (p < 0.05) was clear than the CFAA 20 group excepted TG (p > 0.05) and LDLC (p > 0.05) compared to the HFD group.

**Table 1:** Effect of chloroform fraction from *Actinidia arguta* (CFAA) on glutamic oxaloacetic transaminase (GOT), glutamine pyruvic transaminase (GPT), lactate dehydrogenase (LDH), total cholesterol (TCHO), triglyceride (TG) and low density lipoprotein cholesterol (LDLC) in serum.

Group	NC	HFD	CFAA 20	CFAA 40
GOT (U/L)	47.22 ± 3.42^d^	131.33 ± 19.78^a^	113.20 ± 14.02^b^	79.60 ± 13.31^c^
GPT (U/L)	40.66 ± 7.22^d^	264.75 ± 36.83^a^	168.50 ± 27.18^b^	135.00 ± 21.96^c^
LDH (mg/dL)	101.00 ± 8.83^d^	778.00 ± 134.90^a^	522.75 ± 49.03^b^	371.50 ± 25.59^c^
TCHO (mg/dL)	139.00 ± 10.82^d^	291.75 ± 11.52^a^	233.00 ± 35.34^b^	194.40 ± 17.09^c^
TG (mg/dL)	139.25 ± 16.17^ab^	154.00 ± 15.45^a^	144.00 ± 19.91^ab^	132.00 ± 16.30^b^
LDLC (mg/dL)^*^	31.20 ± 1.91^b^	43.70 ± 7.28^a^	31.00 ± 5.26^b^	26.15 ± 10.53^b^

Results shown are mean ± SD (*n* = 7). Data were statistically considered at p < 0.05, and different small letters represent statistical difference.

^*^LDLC (mg/dl) = TCHO - (HDLC + TG/5).

### Cholinergic system in brain

**Figure 5:**
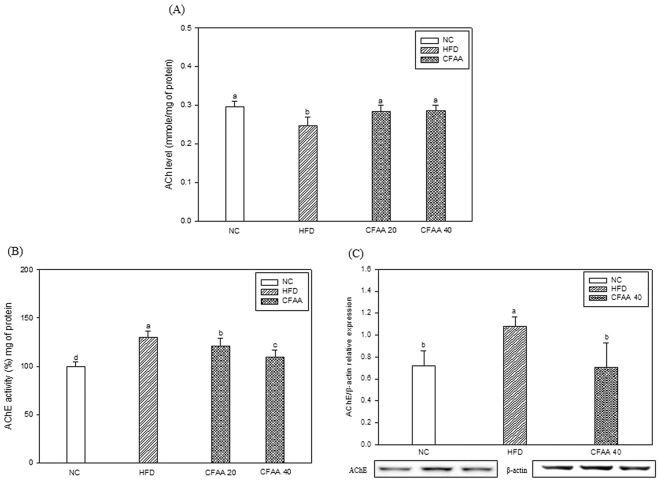
Effect of chloroform fraction from *Actinidia arguta* (CFAA) on acetylcholine (ACh) level (**A**), acetylcholinesterase (AChE) activity (**B**) and the cerebral protein expression level of AChE (**C**) in mice brain. Data were analyzed using ANOVA with Duncan’s SAS and expressed as mean ± SD (*n* = 7). Data were statistically considered at *p* < 0.05, and different small letters represent statistical difference.

As shown in Fig. 5A, the HFD group showed decreased ACh levels of 0.2478 mmole/mg of protein compared with the NC group (0.2964 mmole/mg of protein, *p* < 0.05). The CFAA groups (20 and 40 mg/kg of body weight) showed similar to ACh content of NC group (*p* > 0.05) and increased ACh levels of 0.2840 (*p* < 0.05) and 0.2860 (*p* < 0.05) mmole/mg of protein compared with HFD group, respectively. In addition, the HFD group showed significantly increased AChE levels of 130.10% compared with the NC group (100.00%, *p* < 0.05), whereas the CFAA groups (20 and 40 mg/kg of body weight) significantly showed decreased AChE levels of 120.70 (*p* < 0.05) and 109.14% (*p* < 0.05), respectively (Fig. 5B). Especially, the AChE levels of CFAA 40 group was more decreased than CFAA 20 group (*p* < 0.05). Likewise, with western blotting, AChE expression in the HFD group showed increased levels of 1.0784 compared with the NC group (0.7196, *p* < 0.05), whereas the CFAA 40 group showed AChE expression level similar to NC (*p* > 0.05) and reversed AChE expression levels of 0.7048 in comparison with the HFD group (*p* < 0.05) (Fig. 5C).

### Antioxidant system in brain and liver tissues

**Figure 6:**
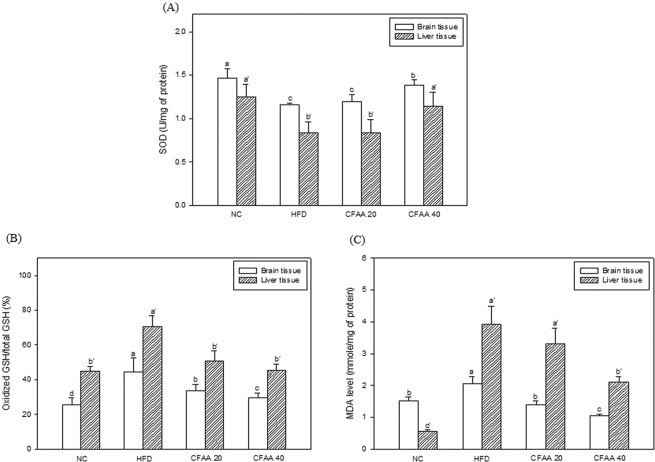
Effect of chloroform fraction from *Actinidia arguta* (CFAA) on superoxide dismutase (SOD) level (**A**), oxidized glutathione/total glutathione ratio (**B**) and malondialdehyde (MDA) level (**C**) in mice brain and liver tissues. Data were analyzed using ANOVA with Duncan’s SAS and expressed as mean ± SD (*n* = 7). Data were statistically considered at *p* < 0.05, and different small letters represent statistical difference.

The effect of CFAA on SOD in brain and liver is shown as in Fig. 6A. The HFD group showed decreased SOD levels of 1.1586 and 0.8398 U/mg of protein compared with the NC group (1.4696 and 1.2498 U/mg of protein, *p* < 0.05), respectively. The CFAA 20 group showed no statistical difference compared with the HFD group in both tissues (*p* > 0.05), whereas the CFAA 40 group showed increased SOD levels of 1.3844 (*p* < 0.05) and 1.1409 (*p* < 0.05) U/mg of protein, respectively.

The results of oxidized GSH/total GSH ratio in the brain and liver is shown in Fig. 6B. The HFD group showed increased ratios of 44.70 and 70.34% compared with the NC group (25.58 and 44.89%, *p* < 0.05), respectively. The CFAA groups (20 and 40 mg/kg of body weight) showed decreased ratios of 33.90 and 50.81% in the 20 group, and 29.63 and 45.16% in the 40 group compared with HFD group (*p* < 0.05).

The results of MDA in the brain and liver are shown in Fig. 6C. The HFD group showed significantly increased MDA levels of 2.0570 and 3.9179 mmole/mg of protein compared with the NC group (1.5255 and 0.5637 mmole/mg of protein. *p* < 0.05). The CFAA 20 group showed no statistical difference in the liver compared with the HFD group (*p* < 0.05), whereas the CFAA 20 group showed significant MDA levels in the brain, similar to the NC group (*p* > 0.05). The CFAA 40 group showed all of decreased MDA levels of 1.0477 and 2.0979 mmole/mg of protein in comparison with the HFD group (*p* < 0.05).

### Mitochondrial dysfunction

**Figure 7:**
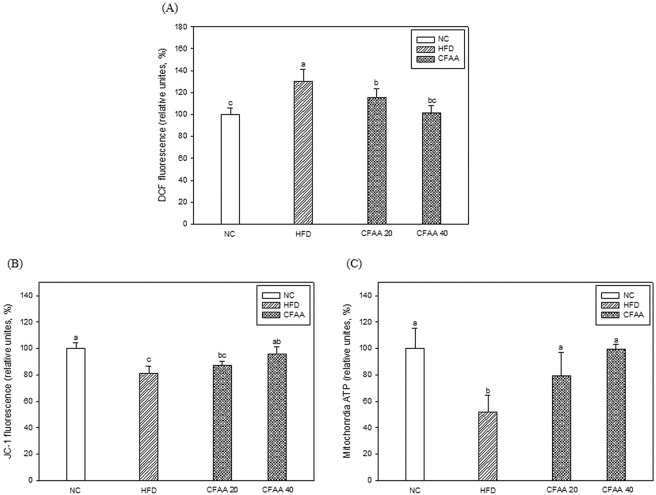
Effect of chloroform fraction from *Actinidia arguta* (CFAA) on mitochondrial ROS production (**A**), mitochondrial membrane potential (MMP) (**B**) and mitochondrial ATP content (**C**). Data were analyzed using ANOVA with Duncan’s SAS and expressed as mean ± SD (*n* = 5). Data were statistically considered at *p* < 0.05, and different small letters represent statistical difference.

ROS levels such as DCF concentrations, MMP, and ATP content were investigated to estimate the effect of CFAA on the abnormal activity of mitochondria induced by HFD. As shown in Fig. 7A, the ROS levels of the HFD group (130.13%) were highest, followed by the CFAA 20 and 40 groups, at 115.35 and 101.47% (*p* < 0.05), respectively, in comparison with the NC group (100.00%) (*p* < 0.05). Regarding MMP levels, the CFAA 40 group (95.89%) was significantly higher than the HFD group (81.23%, *p* < 0.05) when compared with the NC group (100.00% *p* > 0.05) (Fig. 7B). In contrast, the MMP levels tended to increase in the CFAA 20 group, but not significantly different from the HFD group (*p* > 0.05). The contents of ATP were lowest in the HFD group (52.13%), and the CFAA 20 and 40 groups increased to 79.26 (*p* < 0.05) and 99.23% (*p* < 0.05) compared with HFD group, respectively (Fig. 7C).

### Western blot analysis

**Figure 8:**
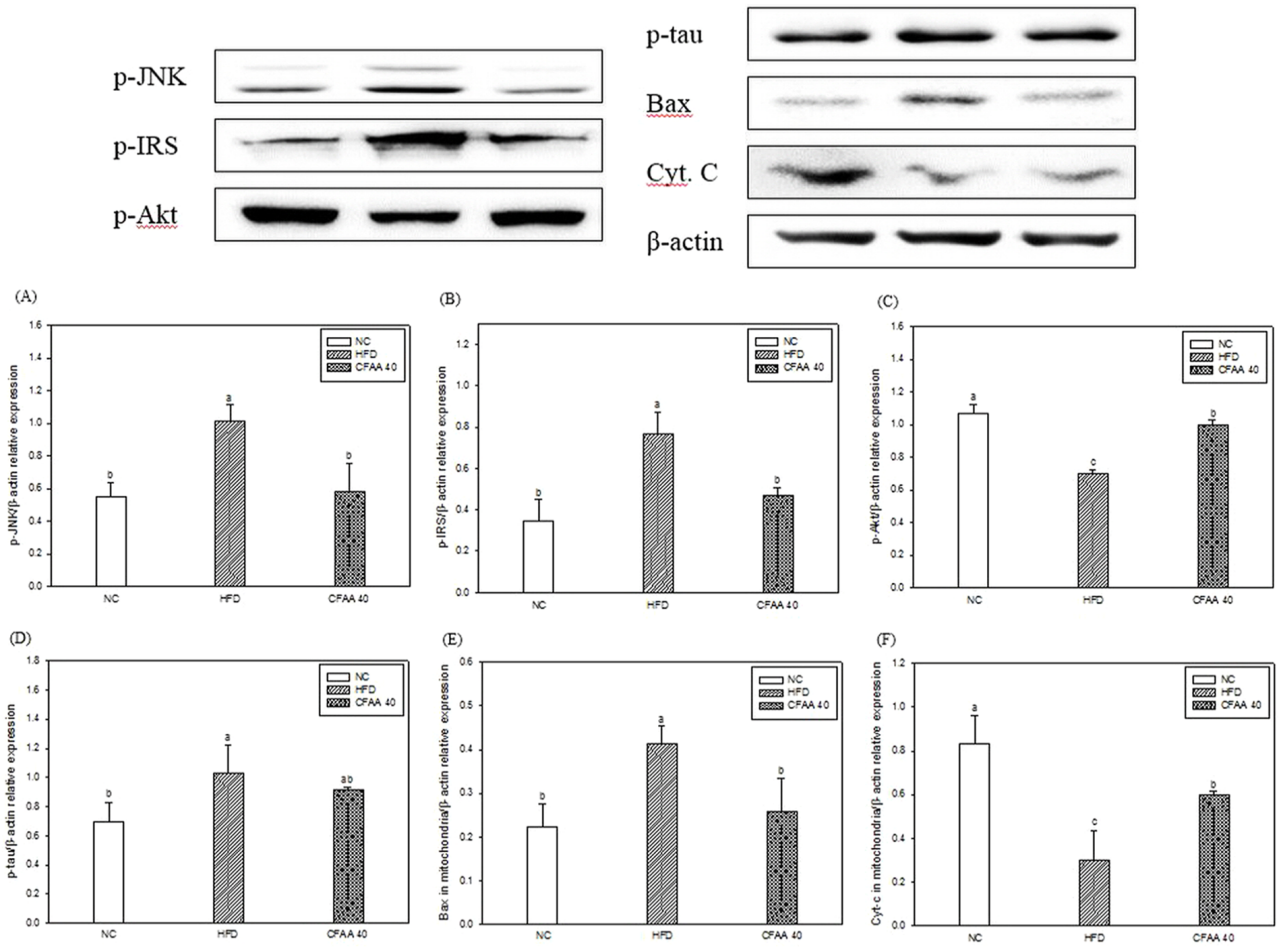
The cerebral protein expression levels of p-JNK (**A**), p-IRS (**B**), p-Akt (**C**), p-tau (**D**), Bax (**E**) in the mitochondria and cytochrome C in the mitochondria (**F**) from HFD-induced diabetic mice. Data were analyzed using ANOVA with Duncan’s SAS and expressed as mean ± SD (*n* = 6). Data were statistically considered at *p* < 0.05, and different small letters represent statistical difference.

The results of western blot assay for protein expression in brain tissue are shown in Fig. 8. In the HFD group, the p-JNK (relative density: 1.014), p-IRS (relative density: 0.765), p-tau (relative density: 1.026) and Bax (relative density: 0.412) increased, and p-Akt (relative density: 0.696) and cytochrome C in mitochondria (relative density: 0.301) decreased compared with the NC group (*p* < 0.05) [p-JNK (relative density: 0.553), p-IRS (relative density: 0.348), p-tau (relative density: 0.692) and Bax (relative density: 0.224), p-Akt (relative density: 1.066) and cytochrome C in mitochondria (relative density: 0.833)]. On the other hand, the CFAA 40 group [p-JNK (relative density: 0.579, *p* < 0.05), p-IRS (relative density: 0.469, *p* < 0.05), p-tau (relative density: 0.916, *p* > 0.05) and Bax (relative density: 0.259, *p* < 0.05), p-Akt (relative density: 0.996, *p* < 0.05) and cytochrome C in mitochondria (relative density: 0.596, *p* < 0.05)] showed tendency to improve compared with HFD group. In other words, CFAA inhibited protein expression change due to HFD.

### UPLC Q-TOF/MS analysis

**Figure 9:**
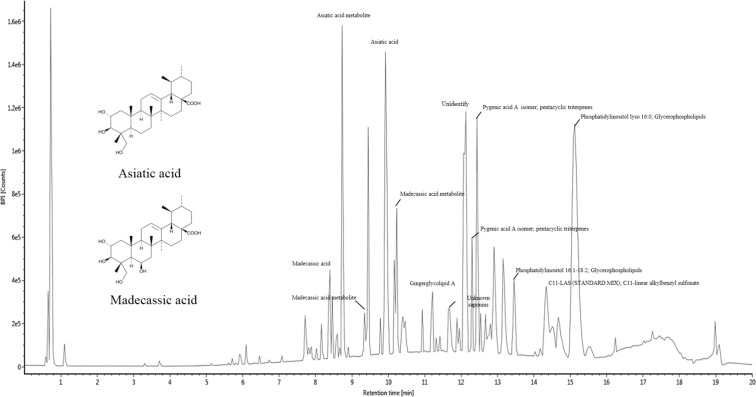
UPLC Q-TOF/MS chromatography in negative ion mode of chloroform fraction of *Actinidia arguta* (CFAA).

Figure 9 and Table 2 show the main compounds of CFAA. The compounds were identified using the Massbank database (https://massbank.eu) and previous research^13,14^. The main compounds were identified as asiatic acid (AA) and metabolites of AA. In addition, we identified pygenic acid A isomer, madecassic acid (MA), metabolites of MA and glycerophospholipids. The AA, MA and pygenic acid A isomer are all pentacyclic triterpenoids. That is, we confirmed that CFAA is rich in pentacyclic triterpenoids.

**Table 2:** Identification of main compounds in the chloroform fraction of *Actinidia arguta* (CFAA).

No.	RT (min)	[M-H]- (m/z)	MS2 fragments (m/z)	Proposed compound
1	8.39	503.34	485.32, 453.29	Madecassic acid
2	8.73	503.34	485.32	Asiatic acid metabolite
3	9.34	501.32	483.31	Madecassic acid metabolite
4	9.45	485.32	485.32	Asiatic acid metabolite
5	9.92	487.34	487.34	Asiatic acid
6	10.23	487.34	469.32	Madecassic acid metabolite
7	11.21	721.37	277.21, 397.13, 415.14, 235.08	Gingerglycolipid A
8	11.68	593.28	315.04, 277.21, 152.99	Unknown saponins
9	12.13	559.31	277.21	Unidentify
10	12.27	471.35	471.34	Pygenic acid A isomer; pentacyclic triterpenes
11	12.43	471.35	471.34	Pygenic acid A isomer; pentacyclic triterpenes
12	13.45	595.29	279.23, 241.00	Phosphatidylinositol 16:1–18:2; Glycerophospholipids
13	14.34	311.16	183.01, 119.05	C11-LAS (STANDARD MIX); C11-linear alkylbenzyl sulfonate
14	15.12	571.29	255.23, 241.01, 152.99	Phosphatidylinositol lyso 16:0; Glycerophospholipids

## Discussion

It had been reported that obesity induces oxidative stress and contributes to the development of type 2 diabetes and insulin resistance, and is involved in the pathogenesis of AD^3^. In addition, it was reported that problems such as HFD-induced cytokines, increased oxidative stress and adipocytes impair the insulin action associated with neuronal synaptic plasticity, and rodents fed HFD for a long time can have cognitive deficits due to brain inflammation, oxidative stress and insulin resistance^15,16^.

In the *in vitro* cell test, CFAA was effective in protecting HG-induced neurotoxicity (Fig. 1). The generation of HG-induced ROS is a well-described mechanism and principally leads to the apoptosis of neuronal cells^17^. Furthermore, the membrane of neuronal cells is vulnerable to attacks by ROS because it consists of a structure of polyunsaturated fatty acids such as linoleic acid and arachidonic acid^18^. For this reason, in our experiments, it was also confirmed an an increase in ROS production and release of LDH, and thereby reduced cell viability in the HG group (Fig. 1). CFAA was effective in protecting HG-induced neurotoxicity, and the protection effect of CFAA is presumed to be related to the antioxidant activity of CFAA. Compared with the vitamin C group, CFAA has similar effects to vitamin C, which is well known as an antioxidant. According to Lim *et al*.^19^, various polyphenols such as protocatechuic acid, caffeic acid and quercetin were analyzed in the fruits of *A. arguta*, and these compounds showed antioxidant and anti-inflammatory effects.

In an animal experiment, it is considered that the mass consumption of HFD contribute to insulin resistance where cells fail to respond normally to the hormone insulin. The improved glucose tolerance and decreased fasting glucose levels in the CFAA groups (Fig. 2) may be related to the α-glucosidase and α-amylase inhibitory activity of CFAA (supplementary material; Figure S1). It is reported that the inhibition of α-glucosidase and α-amylase can significantly reduce the postprandial increase in blood glucose, and therefore can be an important strategy in the management of blood glucose level in type 2 diabetic and borderline patients^9^. The long-term ingestion of acarbose, which is an anti-diabetes drug, may reduce glucose toxicity and control hyperglycemia, but adverse reactions including abdominal pain and diarrhea have been reported^20^. Because of these side effects, the study of natural materials with α-glucosidase and α-amylase inhibitory activity is essential for the improvement of diabetes. According to Kurakane *et al*.^8^, the polyphenols of *A. arguta* have been shown to inhibit the progression of diabetes by enhancing glucose tolerance in type 2 diabetic mice with the inhibition of α-glucosidase. The results of serum analysis also demonstrated improved glucose tolerance in the CFAA group. Excessive elevation of GOT and GPT, which is well known as a biomarker for liver dysfunction, can cause dyslipidemia by causing hypermetabolism of lipids such as cholesterol^21^. In addition, Basciano *et al*.^22^, reported that the insulin resistance caused by hyperglycemia leads to dyslipidemia, which is characterized by hypertriglyceridemia, decreased high-density lipoprotein (HDL) cholesterol levels, and increased low-density lipoprotein (LDL) cholesterol levels. In this experiment, it was observed that GOP and GTP was decreased and dyslipidemia was improved in the CFAA group (Table 1), and these results indicate that insulin resistance was improved in the CFAA group.

In the test of cognitive function evaluated by the three behavior tests, it was confirmed that the cognitive dysfunction of HFD-induced insulin-resistant mice models was decreased, while in contrast, the CFAA groups improved compared with the HFD group (Figs. 3 and 4). It is guessed that there is a relationship between improved glucose tolerance and improved cognitive function in the CFAA group. In addition, the results of brain biochemicals analysis proved that CFAA was effective in improving brain disorders caused by HFD. ACh as a neurotransmitter and AChE as a hydrolase play important roles in signal transfer and signal termination in nerve synapses. The states of decreased ACh level and increased AChE level in the hippocampus and cortex are known to be associated with progressive cognitive decline^23^. According to a previous study^24^, a significant increase in AChE activity was observed in HFD-induced diabetic rats, and prolonged elevation of AChE by HFD may contribute to the acceleration of cognitive decline by reducing the ACh level^25^. In addition, it is reported that plasma and tissue concentrations of butyrylcholinesterase and AChE were elevated in type 2 diabetes and AD^26^. Fig. 5 shows that CFAA significantly increased ACh level and decreased AChE activity and expression. In addition, decreased oxidative stress in the CFAA group may also be associated with improved cognitive dysfunction (Fig. 6). The consumption of HFD leads to increased oxidative stress, which is associated with cognitive disruption such as learning and memory impairments^15^. Furthermore, it is also well known that HG also increases oxidative stress due to insulin resistance^17^. To confirm oxidative stress levels, biochemicals in brain and liver tissues were analyzed. Figure 6A,B shows how CFAA inhibited the down-regulation of SOD and the oxidized GSH/total GSH ratio by HFD-induced oxidative stress in brain and liver tissues. In addition, the level of reduced MDA, which was used as an oxidative stress marker in the CFAA group, clearly showed that oxidative stress was reduced in the CFAA groups (Fig. 6C). The biochemical antioxidants SOD and GSH play an important role in reducing oxidative stress by converting ROS such as superoxide radicals and hydroxyl peroxide into nontoxic molecules^27^. MDA is a marker of lipid peroxidation and causes the impairment of cell the membrane. In addition, it is reported that excessive MDA induced by oxidative stress leads to learning and memory impairments^28^. According to research by Soobrattee *et al*.^29^, an imbalance between oxidant and antioxidant by increasing oxidative stress reduces antioxidant levels, and antioxidants such as phenolics, flavonoids, and vitamin C might help to maintain the homeostasis to SOD and oxidized GSH/total GSH ratio, and suppress increasing oxidative stress. Reduced LDH content in serum can also indicate decreased oxidative stress in the CFAA group (Table 1). LDH, an indicator of tissue damage, is contained in the cells of various tissues, and when the cells are destroyed, they are released into the blood^21^. In summary, it is presumed that the improvement of glucose tolerance due to CFAA and the antioxidant activity of CFAA can effectively suppress the oxidative stress caused by insulin resistance due to HFD, and accordingly CFAA may affect the improvement of cognitive function.

Improved mitochondrial activity can also be evidence that CFAA is implicated in improving cognitive function. Mitochondria play an important role in the control of cellular energy status, ROS production, intracellular calcium metabolism and ATP synthesis, as well as disruption of abnormal cells. In mitochondria, ROS acts as a cellular messenger in redox signaling, whereas excessive ROS induces oxidative stress, which in turn brings about mitochondrial dysfunctions. The distinctive features of mitochondrial dysfunction include some changes such as an elevation of ROS and reduction of MMP. The collapse of MMP induced by oxidative stress triggers the release of cytochrome C, which ultimately activates apoptosis signals and “caspase-3-dependent apoptosis.”^30^ This study showed that HFD induced increased ROS production and decreased MMP and ATP in mitochondria, but the administration of CFAA improved HFD-induced mitochondrial dysfunction (Fig. 7). In addition, it was observed that the release of cytochrome C from mitochondria to cytoplasm decreased and the pathway leading to the release of cytochrome C was also inhibited in the CFAA group (Fig. 8). The c-Jun NH_2_-terminal kinase (JNK) signaling pathway is essential for neuronal apoptosis, and the activation of JNK, which is activated by the phosphorylation of threonine (Thr) and tyrosine (Tyr) residues, diminishes insulin gene expression and interferes with insulin action^31^. According to the research of Hirosumi *et al*.^32^, overexpression of JNK increases IRS-1 serine site phosphorylation instead of IRS-1 tyrosine site phosphorylation, resulting in insulin resistance, and this study reported that insulin signaling is improved in JNK1 knockout mice, and accordingly insulin resistance is significantly suppressed. The phosphorylation of the IRS serine site (Ser307) by JNK1 disrupted the interaction between the catalytic domain of the insulin receptor and the phosphor-tyrosine-binding domain of IRS-1, and it is well known that serine phosphorylation of IRS-1 inhibits insulin signal transduction in a variety of cells^33^. In addition, IRS-1 serine site phosphorylation inhibits PI3K phosphorylation, and subsequently inhibits serine/threonine kinase of Akt. Down-regulated Akt activates GSK-3β and induces amyloid formation and hyperphosphorylation of tau by activated GSK-3β^34^. In addition, the inhibition of Akt leads to an increase in the activity of p53 and Bax, which causes apoptosis. Activation of Bax leads to the release of cytochrome C from mitochondria, and cytochrome C induces apoptosis through caspase^5^^,^^31^. Fig. 8 shows how CFAA improved insulin resistance and inhibited neuronal apoptosis by suppressing the JNK pathway. In addition, the decreased hyperphosphorylation of tau, which plays a vital role in the molecular pathogenesis of AD in the brain by the reduction of JNK was observed in the CFAA group^4^. Eventually, this molecular study demonstrated that CFAA is effective in improving insulin resistance-induced cognitive dysfunction.

The major compounds of CFAA identified by UPLC Q-TOF/MS are AA, MA and metabolites of AA and MA (Fig. 9 and Table 2). AA and MA belong to pentacyclic triterpenes, and pentacyclic triterpenes are widely distributed in many medicinal plants commonly used in traditional medicine for the treatment of diabetes and diabetic complications, and pentacyclic triterpenoids have been found to have a variety of physiological activities, and among them, corosolic acid and gymnemic acids are being marketed as therapeutic agents for the treatment of diabetes^35^. In the research of Liu *et al*.^36^ it was confirmed that AA significantly decreased blood glucose levels and increased serum insulin levels by preserving pancreatic beta cells in streptozotocin-induced diabetic rats. In addition, Hsu *et al*.^37^ reported that MA improved glycemic control and hemostatic imbalance, and in addition, that it lowered lipid accumulation as well as attenuated oxidative and inflammatory stress in diabetic mice. In conclusion, but require additional research on the safety of the sample, these results propose that *Actinidia arguta* rich in pentacyclic triterpenoids may be effective as a preventive matter for a therapeutic strategy to improve neurodegenerative disease caused by HFD.

## Supplementary information

Supplementary Figure S1.
