# Supplementary material for: Pentacyclic triterpenoid-rich fraction of the Hardy kiwi (Actinidia arguta) improves brain dysfunction in high fat diet-induced obese mice
Source: Sci Rep. 2020 Apr 1;10:5788. doi: 10.1038/s41598-020-62810-5 (PMC7113284; doi:10.1038/s41598-020-62810-5)
Supplement: Supplementary file 1 — Supplementary Figure S1. [file 41598_2020_62810_MOESM1_ESM.pdf]

Pentacyclic triterpenoid-rich fraction of the Hardy kiwi (*Actinidia arguta*) improves brain dysfunction in high fat diet-induced obese mice

Jeong Su Ha[1,#], Jin Yong Kang[1,#], Jeong Eun Kang[1], Seon Kyeong Park[1], Jong Min Kim[1], Chul-Woo Kim[2], Sung-Il Oh[2], Uk Lee[2], Dae-Ok Kim[3] and Ho Jin Heo[1,*]

***Corresponding author**: Division of Applied Life Science (BK21 plus), Institute of Agriculture and Life Science, Gyeongsang National University, Jinju 52828, Republic of Korea. Tel: +82 55 772 1907; Fax: +82 55 772 1909. E-mail address: hjher@gnu.ac.kr (H. J. Heo).

[1]*Division of Applied Life Science (BK21 plus), Institute of Agriculture and Life Science, Gyeongsang National University, Jinju 52828, Republic of Korea*

[2]*Division of Special Forest Products, National Institute of Forest Science, Suwon 16631, Republic of Korea*

[3]*Department of Food Science and Biotechnology, Kyung Hee University, Yongin, 17104, Republic of Korea*

JSH: orengewater@naver.com, JYK: kangjy2132@naver.com, JEK: kjd940225@naver.com, SKP: tjsrud2510@naver.com, JMK: myrock201@naver.com, CWK: futuretree@korea.kr, SIO: osi0919@korea.kr, UL: rich26@korea.kr, DOK: dokim05@khu.ac.kr, HJH: hjher@gnu.ac.kr

[#]Jeong Su Ha and Jin Yong Kang are co-first authors

Supplementary information

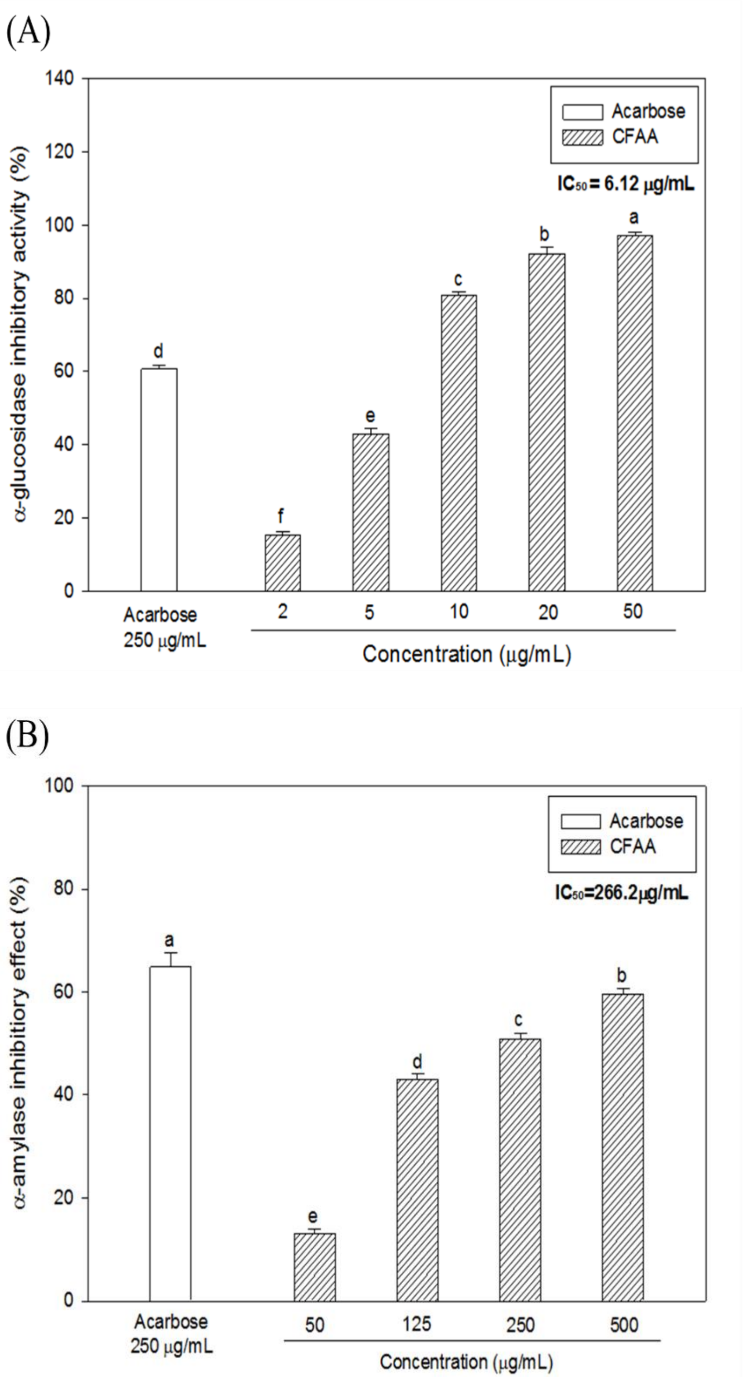

Figure S1. Effect of chloroform fraction from *Actinidia arguta* (CFAA) on α-glucosidase activity (A) and α-amylase activity (B). Data were analyzed using ANOVA with Duncan's SAS and expressed as mean ± SD (n=6). Data were statistically considered at p<0.05, and different small letters represent statistical difference.
